# Determining grasp selection from arm trajectories via deep learning to enable functional hand movement in tetraplegia

**DOI:** 10.1186/s42234-020-00053-5

**Published:** 2020-08-25

**Authors:** Nikunj Bhagat, Kevin King, Richard Ramdeo, Adam Stein, Chad Bouton

**Affiliations:** 1 Feinstein Institutes for Medical Research at Northwell Health, Manhasset, NY USA; 2 Institute for Bioelectronic Medicine, Feinstein Institutes for Medical Research, Manhasset, NY USA; 3 Zucker School of Medicine at Hofstra/Northwell, Hempstead, NY USA

**Keywords:** Spinal cord injury, Neuromuscular stimulation, Inertial measurement unit, IMU, Machine learning, Neural networks, Wearable

## Abstract

**Background:**

Cervical spinal cord injury severely affects grasping ability of its survivors. Fortunately, many individuals with tetraplegia retain residual arm movements that allow them to reach for objects. We propose a wearable technology that utilizes arm movement trajectory information and deep learning methods to determine grasp selection. Furthermore, we combined this approach with neuromuscular stimulation to determine if self-driven functional hand movement could be enabled in spinal cord injury participants.

**Methods:**

Two cervical SCI participants performed arbitrary and natural reaching movements toward target objects in three-dimensional space, which were recorded using an inertial sensor worn on their wrist. Time series classifiers were trained to recognize the trajectories using either a Dynamic Time Warping (DTW) algorithm or a Long Short-Term Memory (LSTM) recurrent neural network**.** As an initial proof-of-concept, we demonstrate real-time classification of the arbitrary movements using DTW only (due to its implementation simplicity), which when used in combination with a high density neuromuscular stimulation sleeve with textile electrodes, enabled participants to perform functional grasping.

**Results:**

Participants were able to consistently perform arbitrary two-dimensional and three-dimensional arm movements which could be classified with high accuracy. Furthermore, it was found that natural reaching trajectories for two different target objects (requiring two different grasp types) were distinct and also discriminable with high accuracy. In offline comparisons, LSTM (mean accuracies 99%) performed significantly better than DTW (mean accuracies 86 and 83%) for both arbitrary and natural reaching movements, respectively. Type I and II errors occurred more frequently for DTW (up to 60 and 15%, respectively), whereas it stayed under 5% for LSTM. Also, DTW achieved online accuracy of 79%.

**Conclusions:**

We demonstrate the feasibility of utilizing arm trajectory information to determine grasp selection using a wearable inertial sensor along with DTW and deep learning methods. Importantly, this technology can be successfully used to control neuromuscular stimulation and restore functional independence to individuals living with paralysis.

**Trial registration:**

NCT, NCT03385005. Registered September 26, 2017

## Introduction

In the United States alone, every year there are more than 17,700 new cases of spinal cord injury (NSCISC 2019). A majority of these injuries results in incomplete (48%) and complete (12%) tetraplegia, which severely affects arm and hand movements of the survivors and undermines their quality of life. Neuromuscular stimulation offers a viable solution to assist with arm and hand movements to increase independence, but often users find it challenging to efficiently control such stimulation devices for everyday use. Therefore, several different modalities have been developed to extract user intent for controlling neuromuscular stimulation devices in order to restore grasping. These modalities range from conventional push button or shoulder position control (Ragnarsson 2008; Cornwall and Hausman 2004), to implanted muscle sensors (Kilgore et al. 2008), and most recently brain implants (Bouton et al. 2016; Ajiboye et al. 2017).

Grasping an object is often preceded by reaching for the object. In fact, previous studies have shown that grasping intentions of amputees and able-bodied participants could be inferred from their muscle activity (electromyogram signals) during reaching (Batzianoulis et al. 2018). Tetraplegia is most often caused by damage to the C5 vertebra and importantly, individuals with C5 and below level injury retain sufficient control over their deltoid and biceps muscles, which allows them to reach for objects (Nas et al. 2015; Prasad et al. 1999). Therefore, we proposed to develop a non-invasive approach that can determine grasp choice in tetraplegia from a user’s natural reaching and also arbitrary arm trajectories. Unlike previous studies that used multi-channel surface electromyography for classifying reaching movements (Batzianoulis et al. 2018), here we used a low-cost, wearable and easy-to-setup inertial sensor. Further, we combined our trajectory recognition algorithms with a custom-built neuromuscular stimulator to determine if functional movement could be achieved in tetraplegic SCI participants.

In recent years, inertial measurement units (IMU) are extensively being used for human computer interactions, particularly for gesture recognition and wearable sensing (Siddiqui and Chan 2020). With advancement in portable computing devices, sophisticated machine learning algorithms such as recurrent neural networks, can be readily deployed for deciphering IMU data (Kim et al. 2019). For an in-depth review on neuromuscular stimulator and inertial sensing for upper-limb movements, we refer the interested reader to several review papers discussing these technologies (Ragnarsson 2008; Popović 2014; Wang et al. 2017; Filippeschi et al. 2017). In this study, we compared a well-known pattern recognition algorithm called Dynamic Time Warping (DTW) with a recurrent neural network for time series classification called Long Short-Term Memory (LSTM). With these methods, we classified arbitrary and natural reaching trajectories for different target objects (requiring different grasp types) in three-dimensional space (3D). We hypothesized that arbitrary arm movements could be classified with high accuracy and that natural reaching movements associated with different target objects, and therefore grasp types, would be distinct and discriminable as well. We further hypothesized that although DTW-based techniques are easily deployable and computationally inexpensive, LSTM networks could outperform DTW within sessions and across multiple days since they are well-suited to classifying time-series patterns of variable length and lag (Hochreiter and Schmidhuber 1997).

In Section 2, methods for the paper describing experimental setup, study protocol, and training of machine learning algorithms are presented. Section 3 presents results from offline and online validation of the algorithms, based on data from two SCI participants and discusses its significance.

## Methods

### Participants

Two participants with tetraplegia were recruited for the study after providing informed consent. The study protocols were approved by the Institutional Review Board of Northwell Health (Great Neck, NY). Participant 1 was a 32 year old male, injured 6 years prior, with a C4/C5 ASIA (American Spinal Injury Association) B injury. He participated in 10 sessions (2 h/session), out of which 7 sessions were used to record 2D and 3D arm movement trajectories. During the remaining 3 sessions, grasp selections were decoded online (in real-time) and used to drive a custom neuromuscular stimulator with textile-based electrodes housed in a sleeve (Ciancibello et al. 2019). This in turn allowed the participant to perform functional movements (e.g. eat a granola bar). Participant 2 was a 28 year old male, injured 10 years prior, with a C4/C5 ASIA A injury. He participated in 3 sessions, which involved 2 training and 1 online testing session.

### Experiment setup and data collection

**Fig. 1:**
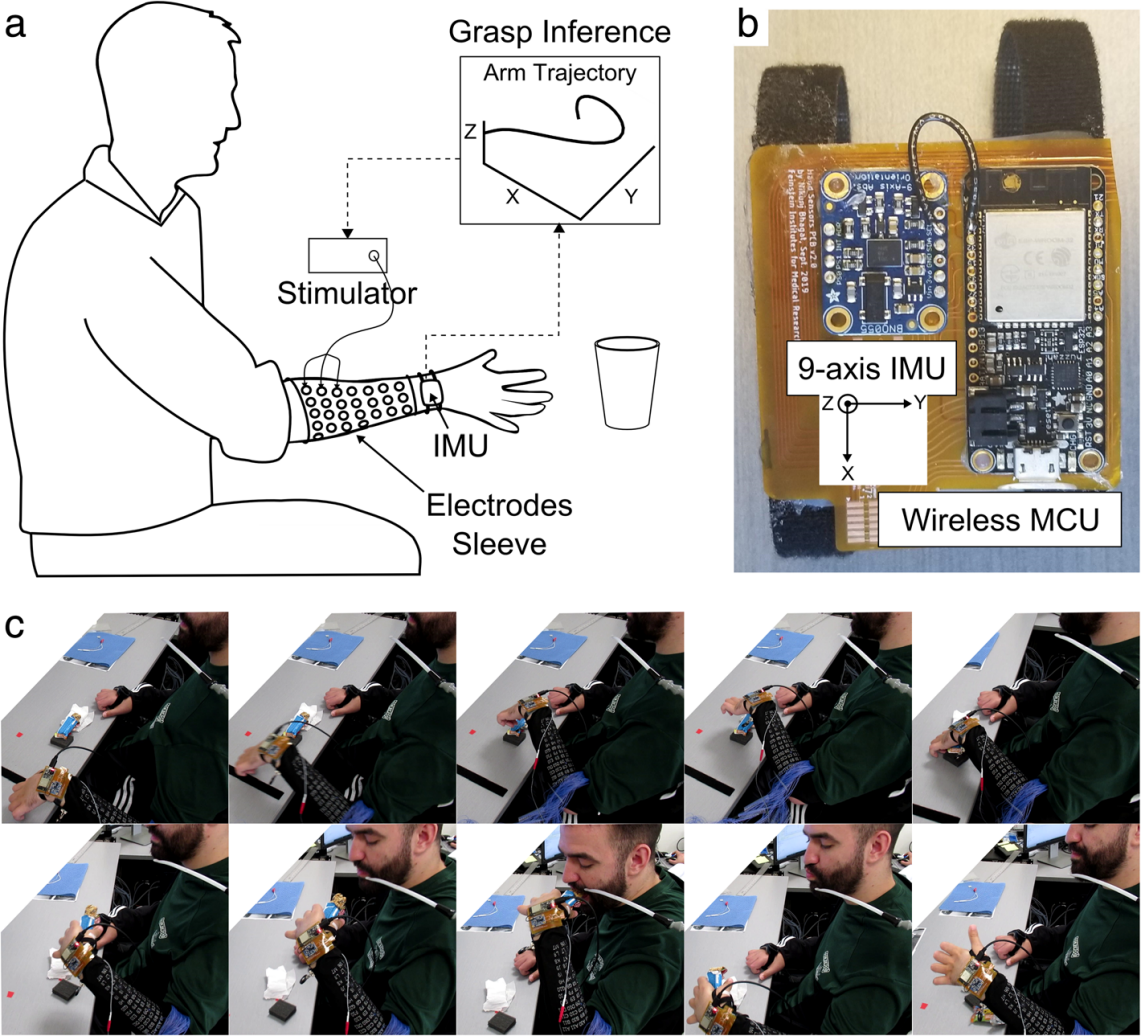
**a** Experimental setup for utilizing reaching movements and enable grasping through the use of an IMU and textile-based electrodes. **b** Closeup of custom-built IMU sensor module with microcontroller (battery not shown). **c** Still images showing an SCI participant using the IMU to activate neuromuscular stimulation and eat a granola bar

Participants were seated with their hands initially resting on a table. A wireless sensor module was attached to the wrist of their arm using a Velcro strap (Fig. 1a). While both participants were bilaterally impaired, each still possessed residual movement that allowed reaching with at least one of their arms, which was then used for the study. The sensor module consisted of a 32-bit ARM microcontroller unit (MCU) from Adafruit (Feather Huzzah32) and a Bosch SensorTec BNO055 9-axis IMU. The IMU has a built-in processor and algorithms to estimate its orientation and perform gravity compensation in real-time to produce linear acceleration in three orthogonal directions. Linear acceleration along the X, Y, and Z axes was available externally via an I2C interface. A flexible printed circuit board was designed to interconnect the IMU with the MCU as shown in Fig. 1b. Data was continuously streamed from the MCU at 50 Hz via Bluetooth to MATLAB 2019a running on a desktop PC and stored for offline processing.

During the experiments, verbal cues associated with arbitrary (2D & 3D) and natural reaching (3D) movement trajectories were randomly called out to the participant. For the arbitrary movements, the participants were instructed to perform the movements within approximately 1 s and to start from a location that allowed them to complete the movement. It is important to note that for the natural reaching trajectories the participants were instructed to reach for two different target objects on the table (a water bottle and a pen), normally associated with two different grasps (cylindrical and pincer/claw grasp respectively), and to stop just in front of the object. Due to their hand impairment, they could not complete the grasping action, but their natural trajectory information was collected to determine if there were two repeatable and discriminable patterns associated with each target object and associated grasp.

The two different natural reaching (3D) movements were called: ‘bottle-reach’ and ‘pen-reach’ (refer to Table 1). Under the arbitrary reaching movement category, four 2D movements and one 3D movement, called corkscrew, were trained. The four 2D trajectories (performed in the horizontal X-Y plane) corresponded to well-known English and Greek letters: S, Ɛ (epsilon or E), γ (gamma), and M. Experiments were conducted in blocks of 18–20 trials and sufficient breaks were given between blocks to minimize participant fatigue. Initially, the participants were asked to perform only S and Ɛ trajectories because these were simple to learn and didn’t cause fatigue. Later, once the participants became comfortable with moving their arm, we included additional 2D trajectories. Thus, in our final datasets there was a higher percentage of 2D trajectories (especially, S and Ɛ) than the remaining trajectories.

**Table 1:**
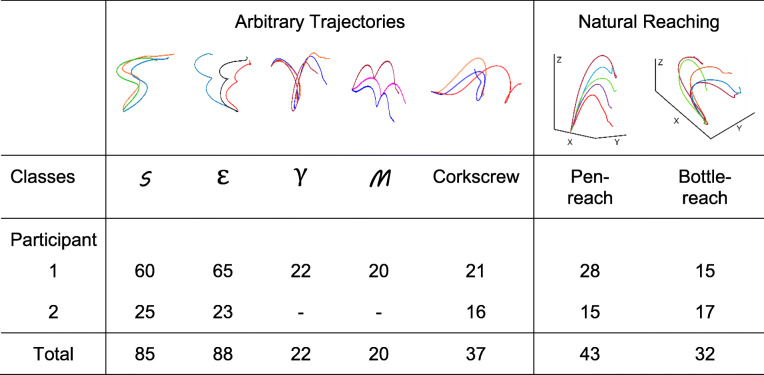
Number of valid 2D and 3D arm trajectories from two participants with tetraplegia, used for training and testing machine learning algorithms. The trajectories are further categorized into arbitrary and natural reaching trajectories in 2D and 3D space

During online (real-time) testing of 2D movement recognition, participants also wore a custom-built fabric sleeve with 128 textile-based electrodes over their forearm to receive neuromuscular stimulation. Neuromuscular stimulation was provided by an 8-channel proprietary, battery-operated, voltage-controlled stimulator. The stimulation parameters were set at 500 μs pulse-width, 20 Hz pulse frequency, and 0 - 110 V output. Additional details of the stimulator design will be presented in a forthcoming publication. The stimulation channels were mapped to individual or multiple electrodes on the fabric sleeve, in order to evoke various finger flexion and extension type movements. The mapping process itself is described in detail in a previous paper (Ciancibello et al. 2019). By grouping multiple stimulation channels and sequencing their activation profile, we could program different hand movements such as opening and closing (and different grasp types).

In a proof-of-concept demonstration in Fig. 1c, an SCI participant uses a single 2D trajectory (M) to initiate a neuromuscular stimulation sequence, which allowed the participant to grasp and eat a granola bar, with his paralyzed hand. The stimulation sequence included the appropriate spatial electrode pattern for opening the hand for 5 s, followed by the pattern to evoke a cylindrical grasp for 5 s to hold the granola bar. Although not implemented here, a second trajectory (e.g. corkscrew) could be used to replace the object back onto the table. The durations for opening and grasping of the hand were selected, to allow enough time for the participant to place his hand around the object and feed himself.

### Data processing and machine learning

The 3-axis linear acceleration obtained from the IMU was band-pass filtered (Butterworth, 8th order, 0.2 – 6 Hz) and processed offline for identifying training samples. The magnitude of the 3-axis acceleration vector was used to identify onset of movement by setting a threshold of 0.95 g. The movement onsets were then used to segment the acceleration data over time along the X, Y, and Z axes into windows ranging − 0.1 s to 0.9 s with respect to onset. Each trial was visually confirmed to be free from any noise artifacts or if it exceeded the 1 s window and such trials were excluded from further analysis. Next, to determine how discriminative the 2D and 3D trajectories were, two time series classifiers based on either a Dynamic Time Warping (DTW) distance measure or Long Short Term Memory (LSTM) network algorithms were trained separately for 2D and 3D trajectories. Each classifier was trained separately for each participant and within each participant, we used a 5-fold stratified cross-validation approach to determine the classifier’s accuracy. For each fold a new classifier model was trained from scratch (nested cross-validation), in order to obtain an unbiased estimate of the classifier’s performance. Further, for statistical comparisons, we combined the classification accuracies from all the folds of both participants, in order to increase the sample size and demonstrate generalizability of the classifiers across participants.

The DTW algorithm optimally aligns a sample trajectory with respect to a previously determined template trajectory such that the Euclidean distance between the two trajectories is minimized. This is achieved by iteratively expanding or shrinking the time axis until an optimal match is obtained. For multivariate data such as acceleration, the algorithm simultaneously minimizes the distance along the different dimensions using dependent time warping (Shokoohi-Yekta et al. 2017). In our DTW-based classifier, this algorithm was used to compute the optimal distance between a test sample and pre-defined templates associated with the 2D and 3D trajectories. Ultimately, the template with the smallest optimal distance to the test sample, was selected as the classifier’s output. The template for each trajectory type was chosen as the training sample with the least aggregate DTW score (i.e. sum of individual scores) to every other training sample of the same type.

To implement the LSTM network we used MATLAB R2019b’s Deep Learning Toolbox with default values for most parameters. Specifically, an LSTM network comprising of a single bidirectional layer with 10 hidden units was used. This transformed the 2D or 3D linear acceleration data into inputs for a fully connected layer whose outcome was binary, i.e. 0 or 1. Next, a softmax layer was used to determine the probability of multiple output classes. Finally, the network output mode was set at ‘last’, so as to generate a decision only after the final time step has passed. This allowed the LSTM classifier to behave similarly to DTW and classify trajectory windows. During training of the LSTM network weights, an adaptive moment estimation (ADAM) solver was used with a gradient threshold of 1 and maximum number of epochs of 200. Since all the training and validation data were 1 s long, zero padding was not used.

During real-time classification of arm trajectories, the linear acceleration signals were filtered and processed in real-time using a MATLAB script that looped at 50 Hz. Within the loop, the acceleration data was divided into 1 s long segments with 98% overlap. To demonstrate an initial proof-of-concept, only the DTW-based classifier was implemented, due to its simple and computationally efficient implementation in MATLAB. For online prediction, the incoming acceleration windows were compared with 2D template trajectories of each type and if the optimal distance between trajectories were below 10 units (empirically determined), then a positive decision was made. This would then trigger our custom neuromuscular stimulator to perform a complete movement sequence of opening and closing of the hand.

## Results and discussion

Over 250 training samples across 7 movement trajectories were recorded for participant 1 and 96 samples from 5 movement trajectories were recorded for participant 2. Trials with noisy sensor data or incorrect labels were visually identified and removed from the dataset. Table 1 shows the distribution of samples across different arbitrary and natural reaching trajectories for both the participants. The top row shows a graphical representation of trajectories of each type by reconstructing a participant’s hand position in space, which was obtained by double integration of linear acceleration.

**Fig. 2:**
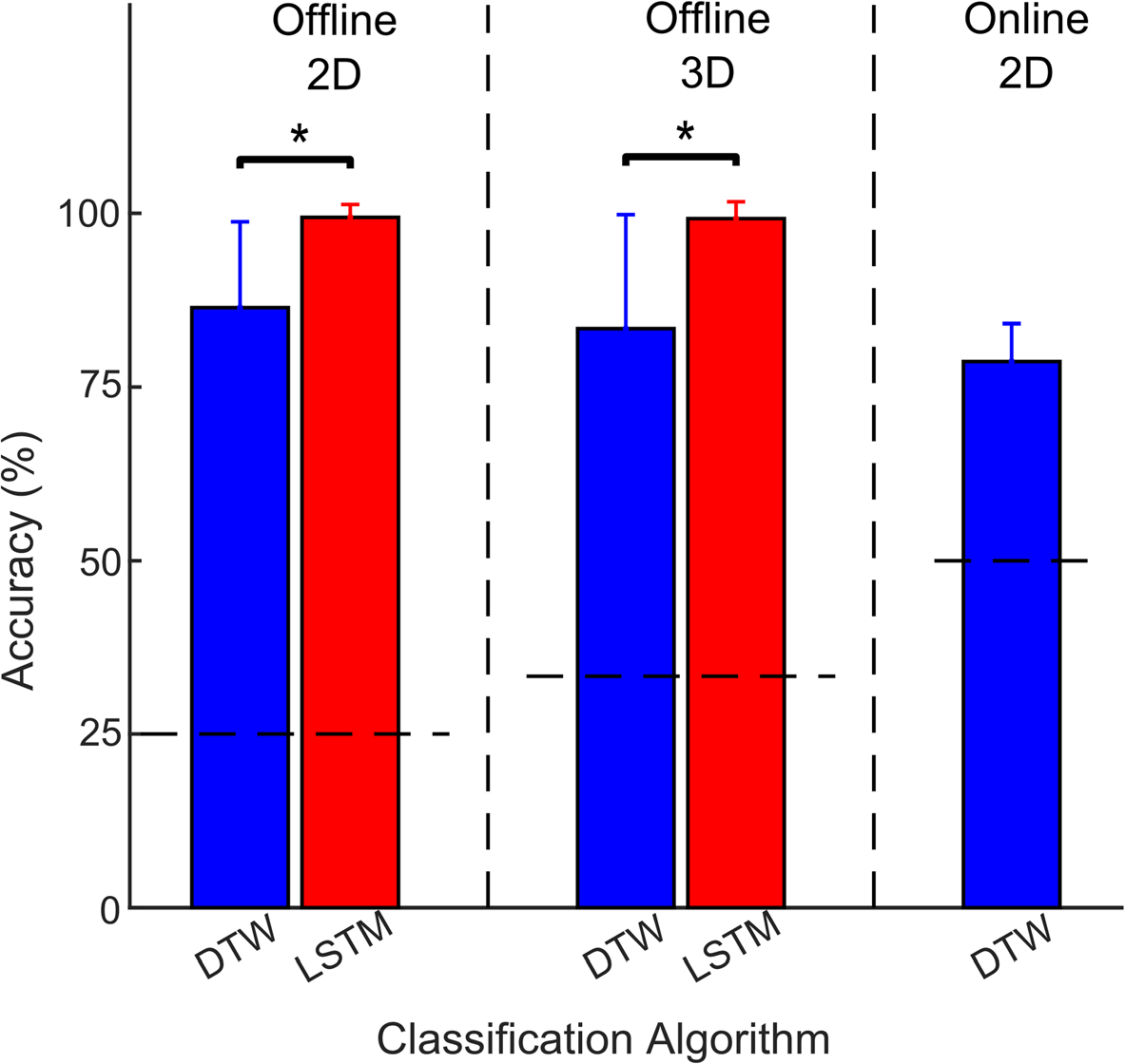
Bar graphs comparing classification accuracies (Mean ± SD) using two machine learning algorithms: DTW and LSTM. Performance was evaluated using both offline (2D & 3D) and online (2D only) arm trajectories. Statistical significance threshold was set at *p* < 0.05. The horizontal dashed lines represents the chance level (= 1/number of classes) under each comparison

**Fig. 3:**
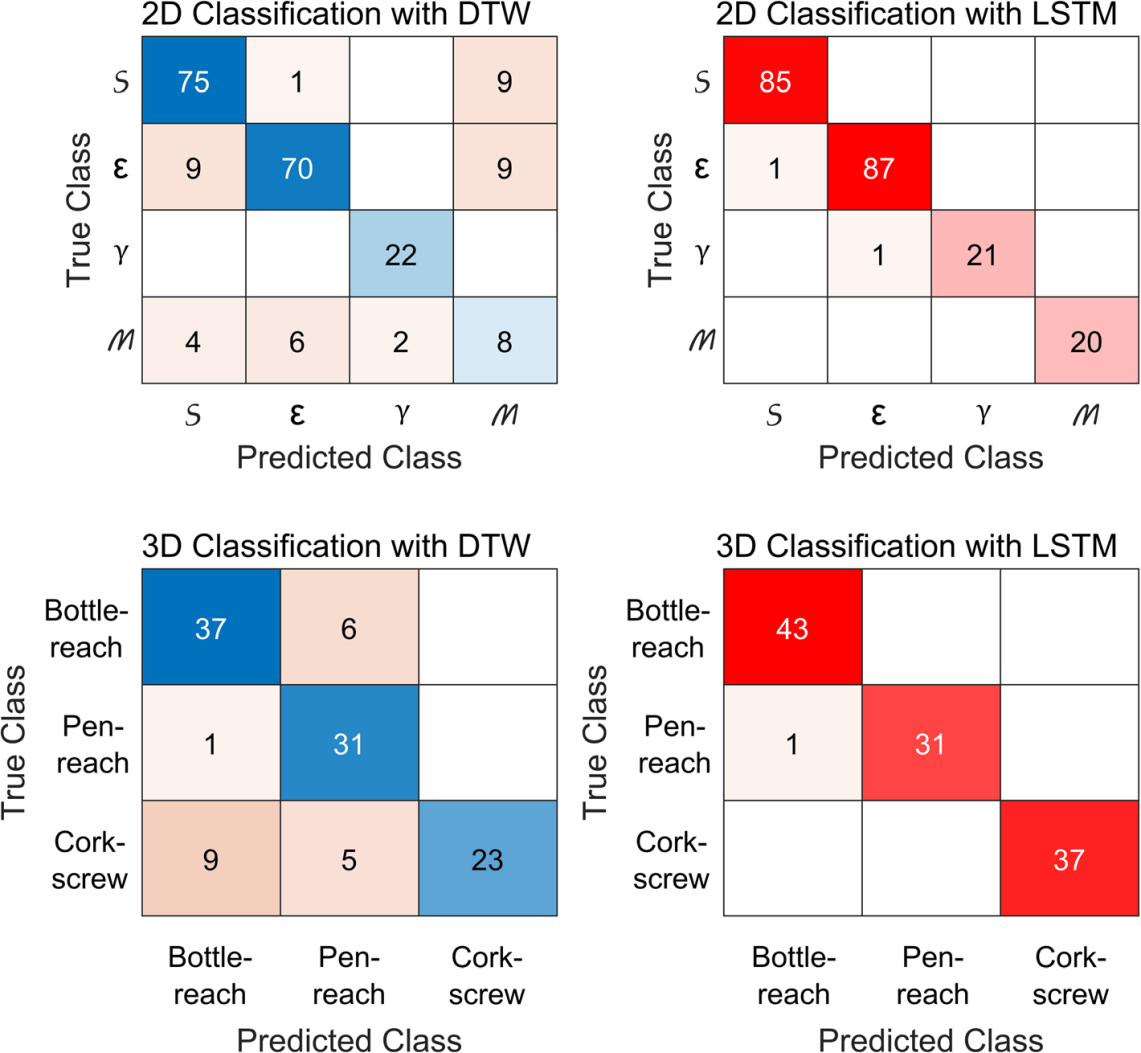
Confusion matrices illustrating DTW and LSTM-based classifier performance for 2D & 3D trajectories

Given the unequal distribution of samples in our dataset, a 5-fold stratified cross-validation scheme was selected for evaluating DTW and LSTM based classifiers. Figure 2 shows the mean ± standard deviation (SD) classification accuracy for the 2 participants. In the offline scenario LSTM performed better than DTW for 2D trajectories, achieving 99 ± 2% (median 100%, IQR 0%) accuracy versus 86 ± 12% (median 89%, IQR 29%), respectively. For 3D trajectories also, LSTM outperformed DTW and obtained 99 ± 2% (median 100%, IQR 0%) accuracy over 83 ± 16% (median 83%, IQR 31%). Using two-sided Wilcoxon signed-rank test, LSTM based classification accuracy was significantly better than DTW for both 2D (*n* = 10, W = 28, *p* = 0.016) and 3D (*n* = 10, W = 21, *p* = 0.0313) movements. Also shown in Fig. 2, is the online performance of DTW based classifier for 2D arbitrary trajectories. During online classification, we either compared between 2 trajectories (e.g. S v/s Ɛ) or between a single trajectory and rest (e.g. M v/s rest) and achieved 79 ± 5% (median 80%, IQR 8%) accuracy. To further evaluate each classifier’s performance for type I and II errors, we calculated their cumulative confusion matrices by combining the confusion matrices from each fold per participant. The resulting confusion matrices for both classifiers and for both types of trajectories are shown in Fig. 3.

For DTW-based classifier, type I error occurred largely for M (60%) trajectory, followed by corkscrew (37.8%) and Ɛ (20.2%) trajectories. In terms of type II errors, DTW-based classifier misclassified bottle-reach (14.5%), pen-reach (13.8%) and S (10%) trajectories more often as compared to rest of the classes. For LSTM-based classifier the type I and II errors were very low and ranged from 0 to 4.5% for all trajectories.

**Fig. 4:**
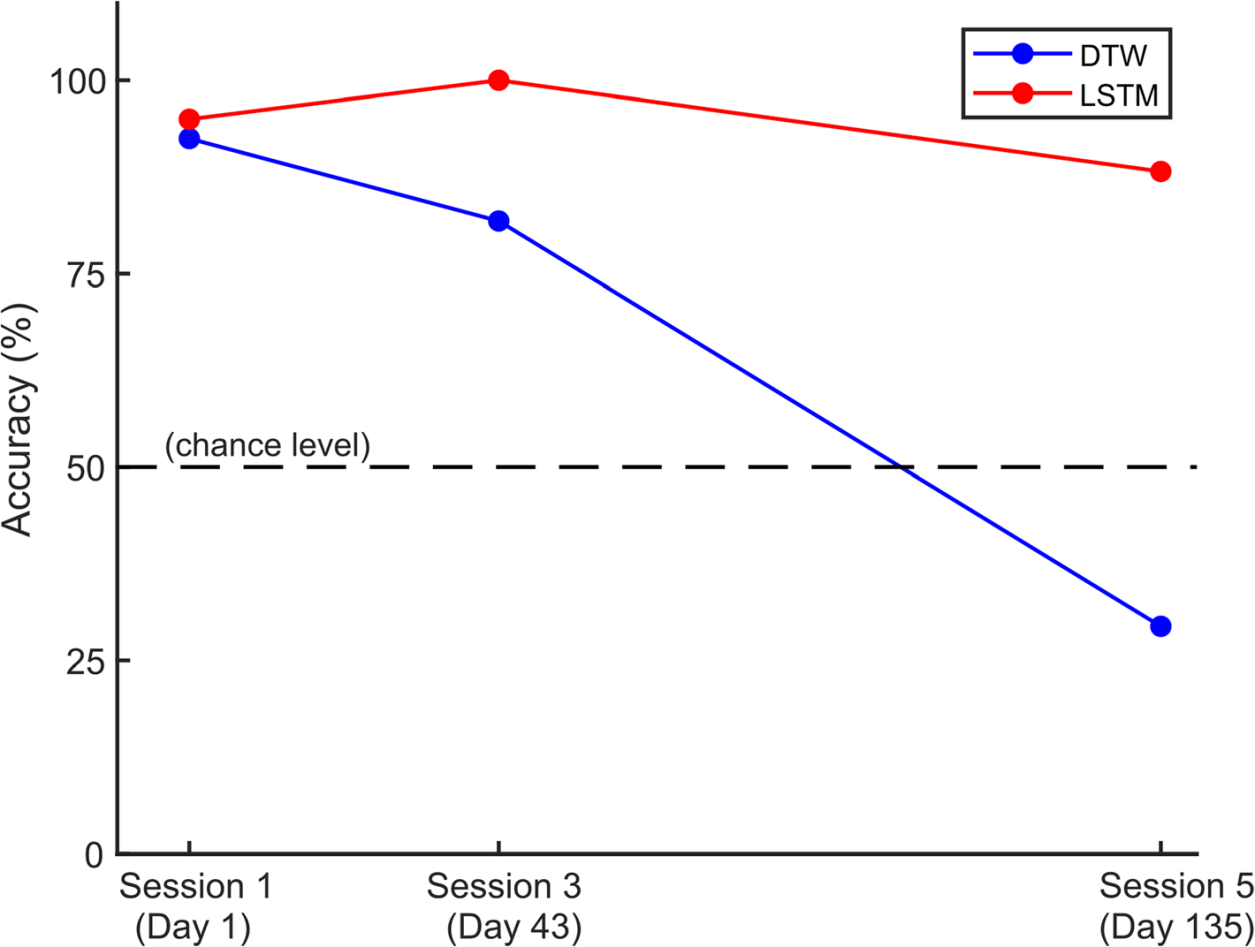
Comparison of LSTM vs. DTW performance across multiple sessions. In this example, data recorded for two 2D trajectories S and Ɛ over three sessions were used for analysis

In order to test the classifiers’ performance across multiple days, we used the existing data for S and Ɛ trajectories from three sessions. Initially, we trained the DTW and LSTM classifiers using only 50% of trials (i.e. 20 trials) from session 1 and then tested them on remaining 50% trials from that session. Next, we used the same model to test trials from sessions 3 and 5 that were recorded several days apart. As shown in Fig. 4, DTW’s performance dropped significantly with time and was below chance level (50%) by session 5. Whereas, LSTM’s performance remained consistently high for all the sessions. This further confirms the superiority of LSTM based classifier over DTW for classifying arm trajectories across multiple days, without requiring frequent recalibrations.

A potential limitation of this study is that the LSTM-based classifier has not been validated during online testing. This is still under development and will be reported in a future publication of this study. Nonetheless, LSTM’s highly robust offline performance, suggests that its online performance will be better than DTW’s online performance. Another limitation is that a reasonable degree of residual arm movements should be preserved in order for the deep learning algorithms to reliably infer grasp intentions. However, given that most tetraplegics include individuals with C5 and lower level injury that retain sufficient arm movements, a majority of SCI survivors will be able to operate this technology. Finally, the current study does not perform an in-depth objective evaluation of grasp performance (e.g. success rate, time to grasp, etc.) or subjective assessment (e.g. ease of use, intuitiveness, etc.) and will be pursued in a follow-up study. Nevertheless, during post-session discussions with the participants, both felt the device was intuitive and easy to learn and utilize.

## Conclusions

This study demonstrates the feasibility of utilizing information from arbitrary and natural arm trajectories to determine grasp selection in tetraplegia. Furthermore, it was shown that machine learning methods can be used to automatically recognize user selections and initiate neuromuscular stimulator patterns for associated grasp types. This approach has clinical viability and could be deployed in rehabilitation centers for use in not only SCI patients, but also individuals living with paralysis from stroke, multiple sclerosis, traumatic brain injury, or other injuries or diseases. Importantly, the rewarding experience of being able to control your own movements, may lead to increased patient engagement during therapy and ultimately, lead to better motor recovery.

## Data Availability

Data from the publication is available from the corresponding author upon request.
